# Less Than Zero? No Worse Memory for Negatively Valued Than Zero Valued Items

**DOI:** 10.1027/1618-3169/a000641

**Published:** 2025-04-23

**Authors:** Ryan P. M. Hackländer, Helge Schlüter, Ann-Kathrin Rolke, Simon Schuster, Christina Bermeitinger

**Affiliations:** ^1^ Department of Psychology, University of Hildesheim, Germany

**Keywords:** forgetting, memory, value directed remembering, recall, recognition

## Abstract

**Abstract:** Not all information encountered is equally important to remember. Some information may be valuable, while others may be irrelevant. Importantly, retrieving and acting upon some information may even have negative consequences. Research has shown that information associated with negative consequences when retrieved is remembered worse than information associated with positive consequences when retrieved. The current experiments address a hitherto understudied aspect of memory for values, namely about how neutral and negative valued information is remembered and which processes underly the encoding and retrieval of this information. Across four experiments, we presented participants with words and an associated positive, neutral, or negative point value. Participants thought the associated values would be added to their total score, thus incentivizing the recall of positive value words and forgetting of negative value words. However, at retrieval participants were told to ignore previously associated values and to try to retrieve as many words from the study phase as possible. Replicating previous research, we found superior retrieval for words associated with positive compared to negative values. More importantly for the current investigation, across four experiments, we found no evidence that words associated with negative values were remembered worse than words associated with a neutral value.


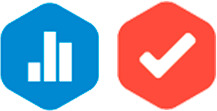


The world is full of information, which humans encounter, encode, and can later retrieve when needed or cued. However, not all information that is encountered is of equal value to remember. While in some cases it is not clear at the time of learning which information may become valuable in the future and which may not (Oberauer & Greve, 2022), at other times it is readily apparent, or there are cues available to make a good approximation as to which information will later be most useful to remember. In such situations, it would be economical if our memory systems were attuned to the consequences of retrieval, and we allocated more resources to encoding and retrieving that information which brings the most valuable consequences.

Research using the value directed remembering paradigm (VDR; Watkins & Bloom, 1998 as cited in Castel et al., 2002) has shown that humans can better remember information that is, at the time of encoding, associated with more positive consequences of retrieval. In the initial publication on the topic, Castel et al. (2002) had participants learn a list of words, each associated with a particular point value that would be earned if the word was remembered (ranging from +1 to +12 points, Experiments 1–3, or ranging from +1 to +20 points, Experiment 4). Free recall tests revealed a graded recall pattern, whereby the higher the point value, the more likely the word would be recalled. The graded retrieval pattern has been replicated many times by the same research group (e.g., Castel et al., 2009, 2011) and other research groups (e.g., Ariel et al., 2015; DeLozier & Rhodes, 2015; Griffin et al., 2019; Murphy & Knowlton, 2022; Park et al., 2022; Robison & Unsworth, 2017; Stefanidi et al., 2018), though not always (e.g., Foster & Sahakyan, 2012, Experiment 3). These retrieval patterns suggest that memory processes are selectively attuned to encode and retrieve information based on how positive the consequences of retrieval are likely to be.

A related paradigm that focuses on value-learning was developed by Madan and Spetch (2012). In this paradigm participants learned, through iterative trials, what face was associated with a particular monetary gain/reward. In a later associative test (value learning test), the authors found some evidence of a U-shaped curve, whereby memory was best for the most positive values, but also where the lowest values were remembered better than the middle values. This pattern seemingly indicates evidence of higher memory for the extremes, rather than a linear increase in memory as a function of associative value. Taken together with evidence from a similar study which also included a proper 0 value at the low end of the reward scale (Castel et al., 2016, Experiment 1), one could interpret these results as evidence of memory being a function of, not only reward value, but also salience.

## Negatively Valued Information

Selection processes must also be active in situations in which some information is associated with positive consequences when retrieved and acted upon, but other information is associated with negative consequences when retrieved and acted upon. In such situations, we could imagine at least two different strategies for the memory system. One strategy would be to allocate encoding and retrieval resources based on the value of the information, with all negatively valued information being allocated no resources. A second strategy would be to invest extra resources into decreasing the probability of retrieval of negatively valued information, perhaps by inhibiting the activation of the information at encoding or retrieval.

A variety of the VDR paradigm has been developed which simulates the presence of positive and/or negative retrieval consequences (Castel et al., 2007). In the study phase participants are presented with words, each of which is followed by a value. The values are either positive or negative and participants are told that they will receive the associated point value if the word is later reported on a memory test. However, before a final memory test, participants are told that the original point values are now irrelevant, and the retrieval of any old word is worth the same number of positive points.

Research using this paradigm has revealed that there is better retrieval of information originally associated with positive values than information originally associated with negative values (Castel et al., 2007; Foster & Sahakyan, 2012; Friedman & Castel, 2011). These findings align with both potential strategies listed above concerning the negatively valued words: (1) Not investing any resources in the negatively valued words or (2) investing resources in actively inhibiting the negatively valued words. Both strategies would lead to worse memory for negatively valued than positively valued words in the experiments mentioned, meaning the experiments are not well suited for determining which of the strategies is more likely to underlie performance on the tests.

## Neutrally Valued Information

In addition to situations in which information is encountered whose retrieval (and the behavior based on that retrieval) leads to lesser or greater, negative or positive, consequences, there are also situations in which retrieval of encountered information, and behaviors based on this retrieval, is not likely to bring either negative or positive consequences. In other words, the information has no, or neutral, consequences when retrieved and acted upon. In such situations, the memory strategies listed above would make different predictions about how information with no consequences upon retrieval should be processed in comparison to information with positive or negative consequences upon retrieval.

If resources were invested selectively as a function of the (positive) value associated with retrieving and reporting the item, then there should be better memory for positively than for neutrally valued information. Investing resources in either negatively or neutrally valued words would be wasteful. This memory strategy, which focuses only on investing mnemonic resources in positively valued information would therefore predict that there should be no difference in resource investment and, accordingly, no difference in retrieval performance between negatively and neutrally valued words.^1^

If resources were invested both in encoding positively valued words *and* in inhibiting negatively valued words, then, like with the previous strategy, there should be better memory for positively than neutrally valued information. However, this strategy would furthermore lead to the prediction that negatively valued information should be remembered *worse* than neutrally valued information. While investing resources in negatively valued information may be economical (i.e., to prevent their retrieval and associated losses associated with acting upon their retrieval in the future), it would be wasteful to invest resources in either encoding or inhibiting neutrally valued information (see Table 1 for an overview).^2^

**Table 1:** An overview of memory strategies, predictions, and supporting evidence

Strategy	Predictions of memory performance as a function of item value	Nature of supporting evidence	References
Invest only in remembering positively valued information	Positive > Neutral = Negative	Equal memory for neutral and negatively valued information	Scholz and Dutke (2019)
Remember positively valued information and inhibit negatively valued information	Positive > Neutral > Negative	Better memory for neutral than negatively valued information	Foster and Sahakyan (2012)

The VDR paradigm has previously been manipulated to also include a neutral condition (i.e., 0 value). In an unpublished Master’s thesis, Lo (2021) failed to find any influence of value (including positive values) on paired-associates memory across three experiments. The lack of the typical VDR effect, whereby positively valued items are remembered better than negatively valued items, makes the nondifference between negatively and neutrally valued items difficult to interpret. Similarly difficult to interpret are findings from a between-subjects study by Foster and Sahakyan (2012, Experiment 4). Here the authors found better recall memory for words with neutral than negative consequences, as would be predicted if a strategy that focused on both encoding words with positive consequences and inhibiting words with negative consequences was implemented. However, the lack of a direct comparison with positively valued words makes it difficult to determine which of the two memory strategies listed above was most likely employed.

The clearest study to date comes from a within-subjects design by Scholz and Dutke (2019, Experiment 2). In this study, participants learned a list of words and each word was associated with one of five possible point values they would receive on a later test (−50, −25, 0, +25, +50). Here the researchers found the typical VDR effect, whereby positively valued words were remembered better than negatively valued words. Furthermore, the researchers found no differences in recognition memory for words with neutral and negative consequences. This finding is most in line with the strategy to only invest resources in remembering positively valued words.

## Current Research

The research on memory for information with neutral or negative consequences of retrieval is both limited and inconclusive to this point. The Lo (2021) and Foster and Sahakyan (2012) results remain difficult to interpret. The Scholz and Dutke (2019) results are clearer, but this is only a single experiment and focused exclusively on recognition memory. Previous research has shown that the test type (i.e., either recall or recognition) has had an influence on both research using the VDR paradigm (see Filiz & Dobbins, 2024 for a discussion) and the similar item method directed forgetting paradigm (e.g., Geiselman et al., 1983). In light of these considerations, the current research set out to investigate two different questions:

*Research Question 1 (RQ 1)*: Is information with negative consequences for retrieval and reporting remembered equally well to, better than, or worse than information with neutral consequences for retrieval?

*Research Question 2 (RQ 2)*: Are any potential differences in memory for information with negative or neutral consequences dependent on the type of item memory test (i.e., recall or recognition) used?

To investigate these questions, we conducted four experiments using either a nongraded VDR paradigm (Experiments 1a & 1b), in which only a single positive and negative value was used, or a graded VDR paradigm (Experiments 2a & 2b), in which different positive and negative values were used, with either a free recall (Experiments 1a & 2a) or an old/new recognition (Experiments 1b & 2b) test. Following the test for item memory, we also included a source memory test in each experiment in which participants had to determine which value each old item was associated with (e.g., Filiz & Dobbins, 2024). In the current paper, we focus exclusively on item-memory performance, given some potential methodological issues with how source-memory was queried. All information about the methods and results of the source memory tests, relevant summary data, as well as a discussion about the potential implications of findings and discussion of methodological concerns can be found on the associated OSF page: https://osf.io/u85vn/.

## Ethics Statement

All experiments were conducted in line with the Declaration of Helsinki (World Medical Association, 2025) and the principles outlined in the Belmont Report. Furthermore, all experiments were approved by the local ethics committee of the Faculty for Education and Social Sciences at the University of Hildesheim (Approval number 216). Across all experiments, participants provided informed consent before participating in the experiment. Following completion of the experiment, all participants were informed about the deception and the reason for the deception. Finally, all participants were provided with contact information of the researchers so that they could ask any questions or report any ethical issues they saw with the experiments.

## Experiments 1a and 1b

On each trial in the study phase of Experiments 1a and 1b, participants were presented with a word and then an associated value (−1, 0, +1). At this stage, participants were led to believe that reporting of the word in the upcoming memory test would lead to the associated points being added (or subtracted) to (or from) their overall score. Before the retrieval phase, participants were told that they should ignore the previously associated values and try to remember as many words as possible (regardless of the previously associated value). In Experiment 1a, participants completed a free recall test, while in Experiment 1b participants completed an old/new recognition test.

### Method

#### Participants

Thirty-two participants volunteered for Experiment 1a. The final sample consisted of 25 females, six males and one nonbinary with a *M*_age_ of 23.5 (*SD* = 4.2; range from 19 to 35) years. 44 participants volunteered for Experiment 1b. Ten participants were excluded from the data analyses for having lower than chance performance on the memory tests. The final sample consisted of 34 participants, 28 females and six males, with a *M*_age_ of 23.1 (*SD* = 5.0; range from 19 to 46) years. Participants for both experiments were recruited using social media, direct contacts, and the university digital study posting board. Participants received either partial course credit or 10€ per hour for participation.

Sensitivity analyses performed with G*Power (Faul et al., 2007) showed that, with the obtained sample sizes, and an α and β level of .05, we would have been able to detect a difference between negative and neutral conditions of an effect sizes of *dz* = .66 (Experiment 1a) and *dz* = .64 (Experiment 1b) using a two-tailed *t* test.

#### Design

For both Experiments 1a and 1b, a one-way design with three levels of the independent variable (Point value: −1, 0, +1) was used. The factor Point value was varied within-subjects. The main dependent variable of interest was accuracy on the retrieval test (Experiment 1a: proportion of words recalled; Experiment 1b: *d*′).

#### Material

Twenty-one words were chosen from the WWN database (Lahl et al., 2009) for Experiment 1a. The words were five letter long nouns with a frequency between 1 and 5 pMW in the German language (Cosmas II), an arousal rating below three on a 10-point Likert scale. 120 words were chosen from the BAWL database (Võ et al., 2009) for Experiment 1b. The words were between 6 and 8 letter long nouns, with a valence rating between −1 and +1 on a Likert scale that ranged from −3 to +3, and an imageability rating above four on a seven-point Likert scale. Word lists can be found on the OSF page: https://osf.io/u85vn/. Experiments were programmed using E-Prime 3.0 software (Psychology Software Tools, 2016).

#### Procedure

Experiments 1a and 1b were completed in the laboratory. Following the provision of consent, participants were informed that we were interested in the strategies that people use to remember relevant information. They were then instructed that they would see a list of words that they should remember for a later memory test. Participants were informed that each word would be associated with a certain point-value (either −1, 0, or +1 points) and that when that word was retrieved and reported, they would *receive* the corresponding number of points. Participants were informed that the goal of the study was to accumulate as many points as possible on the test. The nature of the retrieval test (i.e., recall or recognition) was not known to the participants at this stage.

Following the initial instructions, participants were shown an example of the task and two samples of responses from hypothetical participants. Participants were then asked to determine which of the two hypothetical individuals would receive the most points for their responses. Participants were then provided with feedback concerning the accuracy of their choice, what the correct choice was, and why it was the correct choice. For exact instructions, please see the OSF page: https://osf.io/u85vn/.

##### Study Phase

**Figure 1:**
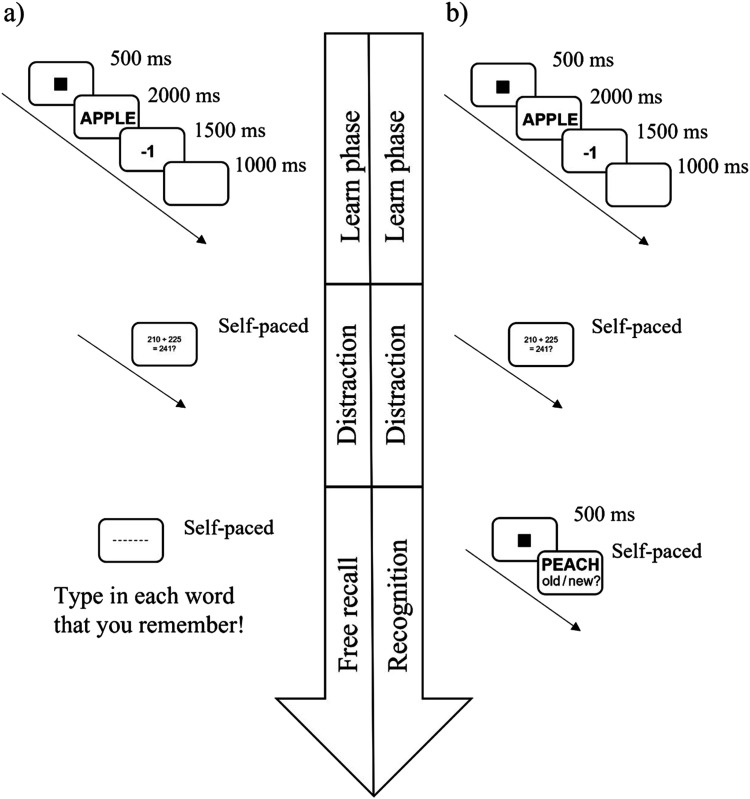
Visualization of the trial procedure in the study phase in Experiments 1a and 1b.

In the study, phase 21 (Experiment 1a) or 60 words (Experiment 1b) were presented to the participants. The words were presented in random order. Each trial began with a fixation symbol (■) presented in the middle of the screen for 500 ms. This was immediately replaced by the target word (presented in 48-point Arial font in black in the middle of the screen on a grey background). After 2,000 ms, the word disappeared and was replaced by the point value (presented in 48-point Arial font in black in the middle of the screen on a grey background), which remained on the screen for 1,500 ms. This was followed by a blank grey screen for 1,000 ms (see Figure 1 for an overview of the trial structure). This sequence repeated until all words were presented.

##### Retention Interval

Participants engaged in a distractor task for at least one minute. Participants saw equations and needed to determine whether the equation was correct (by pressing “m” on the keyboard) or incorrect (by pressing “x” on the keyboard). Participants worked at their own pace and completed as many equations as they could in the time. The distractor task ended with the first response provided 60 s after the beginning of the task.

##### Retrieval Phase

Following the retention interval, participants were instructed to remember the words that they had seen in the study phase. They were now explicitly instructed to remember *all* of the words. Participants were at this point be told that each word was worth +1 point and that the original point values they saw were meaningless.

In Experiment 1a, participants engaged in a self-paced free recall task. Participants typed each word into the program, one at a time, and then pressed *enter* after each word. They continued this process until they could not remember any further words, at which point they pressed enter without writing any word. This ended the recall test.

In Experiment 1b, participants engaged in an old/new recognition task. Each trial began with the fixation symbol being presented for 500 ms, followed by the presentation of a word. On each trial participants had to determine whether the word was old (i.e., was presented in the study phase; response with “j” key) or new (i.e., was not presented in the study phase; response with “f” key). There was no time limit on each trial. 120 words were presented on the recognition test and the 60 words from the study phase and 60 foils. Words were presented in the same font size and on the same background as in the study phase but in Calibri font, due to an oversight in programming. The item memory test was followed by a source-memory test which is not reported here (see the OSF page: https://osf.io/u85vn/).

##### Questionnaires and Debriefing Phase

At the end of the study, participants were asked to answer questions about how they completed the task (see the OSF page: https://osf.io/u85vn/). Afterwards, participants were debriefed about the purpose of the experiment and the need for the deception (i.e., being told that some words had different point values, and then being told that all words were actually worth +1 point during the retrieval phase). Finally, participants were thanked for their participation, provided with contact information of the experimenters and asked to not speak about the experiment with potential further participants.

### Results

#### Data Preparation

Close misspellings, which did not change the meaning of a word, were considered as accurate responses on the recall test in Experiment 1a. The accuracy was rated by SS and RPH independently. There were disagreements in 7 cases. In all cases, agreement was made through discussion. Of the 32 participants, only three answered the question in the practice phase (about which fictional person would receive the most points) incorrectly in Experiment 1a and only two answered the question incorrectly in Experiment 1b. As excluding these participants made no differences in the results, they were included in the analyses reported below. For Experiment 1b, hit rates and false alarm rates were submitted to a log linear transformation (Stanislaw & Todorov, 1999). These transformed rates were used to calculate *d*′.

#### Experiment 1a: Recall

**Figure 2:**
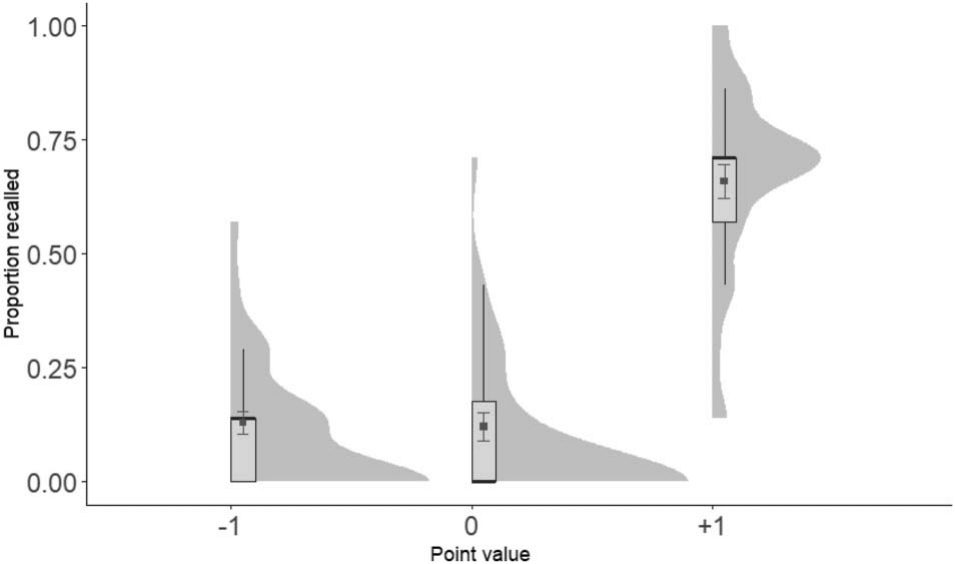
Proportion recalled plotted as a function of Point value in Experiment 1a. Elements include box-plots with medians and density clouds. Squares represent means, and error bars represent ± 1 standard errors. Analyses revealed superior recall for +1 than 0 and −1 point value associated words.

Proportion of recalled words were submitted to a one-way repeated measures ANOVA, with Point value (−1, 0, +1) as the independent variable. The ANOVA revealed a significant main effect of Point value *F*(1.87, 58.01) = 93.08 (Greenhouse-Gesser correction), *p* < .001, η_*p*_^2^ = .75 (see Figure 2). Planned *t* tests showed that +1 words were remembered better than 0, *t*(31) = 11.08, *p* < .001, and −1, *t*(31) = 11.14, *p* < .001, words. There was no difference between the −1 and the 0 words, *t*(31) = .23, *p* = .820 (two-tailed).

#### Experiment 1b: Recognition

**Figure 3:**
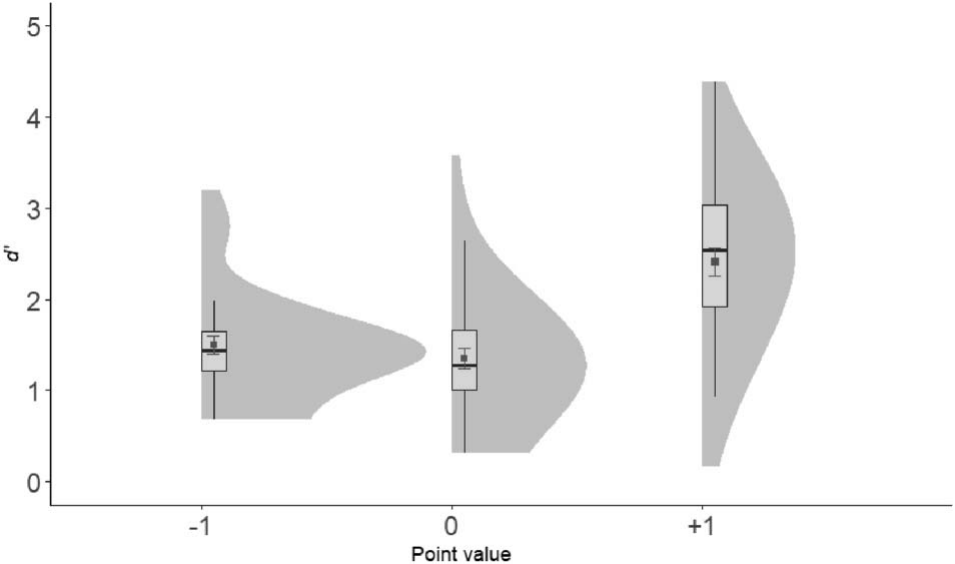
*d*′ plotted as a function of Point value in Experiment 1b. Elements include box-plots with medians and density clouds. Squares represent means, and error bars represent ± 1 standard errors. Analyses revealed higher discrimination for +1 than 0 and −1 point value associated words.

*d*′ values were submitted to a one-way repeated measures ANOVA, with Point value (−1, 0, +1) as the independent variable. The ANOVA revealed a significant main effect *F*(2, 66) = 66.17, *p* < .001, η_*p*_^2^ = .67 (see Figure 3). Planned *t* tests showed that +1 words were remembered better than 0, *t*(33) = 8.47, *p* < .001, and −1, *t*(33) = 9.48, *p* < .001, words. There was no difference between the −1 and the 0 words, *t*(33) = 1.33, *p* = .193 (two-tailed).

### Discussion

In Experiment 1, participants learned a series of words, each associated with one of three possible point values (−1, 0, +1) that would ostensibly be received if the word was later reported on a memory test. Before the test, participants were informed that the previously associated values were not meaningful, and they should attempt to retrieve all the words they learned. The pattern of results from a recall test (Experiment 1a) and a recognition test (Experiment 1b) largely converged.

In line with previous research (Knowlton & Castel, 2022), across both studies, we found that memory for negatively valued words was inferior to memory for positively valued words. While this finding has been replicated several times, it has not always been found (Lo, 2021). This finding is, however, important as a baseline for evaluating the possible effect we were most interested in, namely how negatively valued information would be remembered in relation to neutrally valued information.

In Experiment 1a, there was no difference found between memory for neutrally valued information and negatively valued information. One potential issue with the recall results was the chance of a floor effect, especially for the neutrally and negatively valued words. Performance on the old/new recognition test in Experiment 1b was clearly above chance, and therefore, the worries about a floor effect preventing an interpretation was diminished. As in Experiment 1a, we found no evidence for a difference between memory for neutrally valued information and negatively valued information.

The results of Experiments 1a and 1b best fit an interpretation that a memory strategy was used in which resources were only invested in encoding and retrieving words with a positive value. These findings extend those of Scholz and Dutke (2019), who also found no differences in memory for neutrally and negatively valued information in a recognition test, by finding a similar pattern of results while using a nongraded paradigm and by finding the pattern in both recognition and recall. The results also somewhat match a pattern from Lo (2021) who found no differences in associative memory between neutrally and negatively valued items, though they also found no benefit for positively valued items.

The results are less compatible with a memory strategy that focuses on both strengthening the memory of positively valued items *and* inhibiting the memory of negatively valued items. If such a memory strategy was implemented, we would expect poorer memory for negatively than neutrally valued items (for similar arguments using the directed forgetting paradigm, see Tan et al., 2020).

## Experiments 2a and 2b

While the results of Experiment 1 do not align with a memory strategy that involves inhibiting memory for negatively valued information, it is possible that the design did not allow for enough of a distinction between neutral and negatively valued words. The *negative* value (i.e., −1) may not have seemed particularly negative or different from the neutral value (i.e., 0) to subjects. Increasing the absolute difference from the neutral value may make the negative values seem more negative and may make them more likely to be inhibited. Similarly, adding a graded value structure, with multiple different positive and negative values (e.g., Scholz & Dutke, 2019) may make the negative consequences even more salient, and, again, make it more likely that the (extreme) negatively valued words will be inhibited.

Experiment 2 aimed to include negative values that were further in absolute value from negative values and more likely to be inhibited, if such a memory strategy is utilized. Accordingly, in Experiment 2, participants again learned a list of words associated with different values, but this time there were five different values (−10, −5, 0, +5, +10). The values were now both graded and larger than the values used in Experiment 1 (apart from the neutral value of 0). As in Experiment 1, some participants were tested with a free recall test (Experiment 2a) and others were tested with an old/new recognition test (Experiment 2b).

### Method

#### Participants

Thirty-three participants volunteered for Experiment 2a. Participants were recruited through personal connections and word of mouth by AR for her master’s thesis project. Participants received no monetary renumeration for their participation. Two participants were removed for not having a recall rate of greater than 5%. The final sample consisted of 22 females and nine males with a *M*_age_ of 30.3 (*SD* = 12.0; range from 20 to 60) years.

Forty-six participants volunteered for Experiment 2b. Participants were recruited using social media, direct contacts, and the university digital study posting board. Participants received either partial course credit or 10€ per hour for participation. The sample consisted of 34 females, 11 males, and one diverse individual with a *M*_age_ of 22 (*SD* = 2.8) years.

Sensitivity analyses performed with G*Power (Faul et al., 2007) showed that, with the obtained sample sizes, and α and β levels of .05, we would have been able to detect a difference between the negative and neutral conditions of effect sizes *d*z = .60 (Experiment 1a) and *d*z = .49 (Experiment 1b) using one-tailed *t* tests.

#### Design

For both Experiments 2a and 2b, a one-way design with five levels of the independent variable (Point value: −10, −5, 0, +5, +10) was used. The factor Point value was varied within-subjects. The main dependent variable of interest was accuracy on the retrieval test (Experiment 2a: proportion of words recalled; Experiment 2b: *d*′).

#### Material

Forty words were chosen from the WWN database (Lahl et al., 2009) for Experiment 2a. The words were 5 or 6 letter long nouns with a frequency between 1 and 5 pMW in the German language (Cosmas II), an arousal rating below four on a 10-point Likert scale. 120 words were chosen from the BAWL-R database (Võ et al., 2009) for Experiment 2b (the same as for Experiment 1b), 60 of which were randomly chosen per subject for the study phase and the remaining 60 were distractors on the recognition test (the word lists can be found on the OSF page: https://osf.io/u85vn/). The entire experiment was programmed using E-Prime 3.0 software (Psychology Software Tools, 2016) and was run with E-Prime Go (Psychology Software Tools, 2020).

#### Procedure

**Figure 4:**
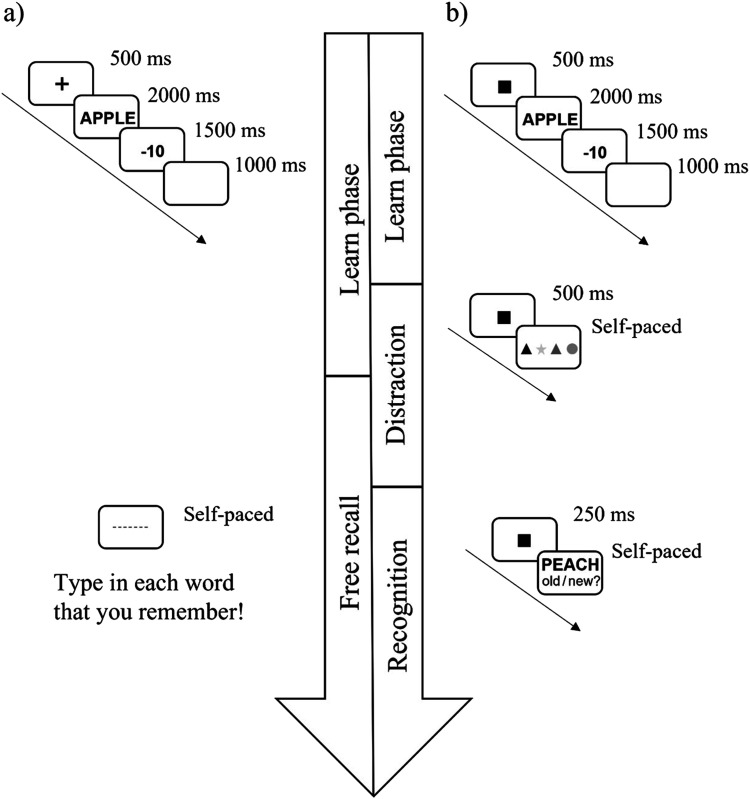
Visualization of the trial procedure in the study phase in Experiments 2a and 2b.

The procedure was identical to that of Experiment 1, with the following exceptions. (1) Each word was associated with one of the following values: −10, −5, 0 +5, +10. Eight words were associated with each possible value in Experiment 2a, and 12 words were associated with each possible value in Experiment 2b. (2) Experiment 2a was completed remotely, by downloading the E-Prime Go package. After receiving information about the study and data protection policies, participants provided informed consent. (3) In Experiment 2a, 40 words were learned in the study phase. (4) In Experiment 2a, a fixation cross was used. (5) In Experiment 2a, there was no retention interval. (6) The nature of the distractor task in the retention interval was different in Experiment 2b than in Experiment 1 (see below). (7) In Experiment 2b, the fixation square was shown for 250 ms in the recognition phase (see Figure 4 for an overview of the trial structure).

##### Retention Interval

In Experiment 2b, participants engaged in a distractor task for two minutes. Participants saw colored shapes on the screen and needed to determine whether all the shapes and colors were unique or whether one repeated. There were four possible colors used (Blue, Green, Yellow, and Red) and four possible shapes that were used (a triangle, a circle, a square, and a star). On each trial, there was a 50% chance that the trial contained either a unique array or that one of the colors or shapes repeated. On trials where one feature repeated, there was a 50% chance that the color repeated and a 50% chance that the shape repeated. There were no trials in which both the color and shape repeated. There was no distractor task in Experiment 1a.

### Results

#### Data Preparation

For the recall task in Experiment 2a, close misspellings, which did not change the meaning of a word, were considered as accurate responses. The accuracy was rated by AR and RPH independently. There were disagreements in only 2 cases. In both cases, agreement was made through discussion. Of the 33 participants, only four answered the question in the practice phase (about which fictional person would receive the most points) incorrectly. As excluding these participants made no differences in the results, they were included in the analyses reported below. Two participants were removed for having performance on the recall test of 5% or below^3^. For Experiment 2b, hit rates and false alarm rates were submitted to a log linear transformation (Stanislaw & Todorov, 1999). These transformed rates were used to calculate *d*′.

#### Experiment 2a: Recall

**Figure 5:**
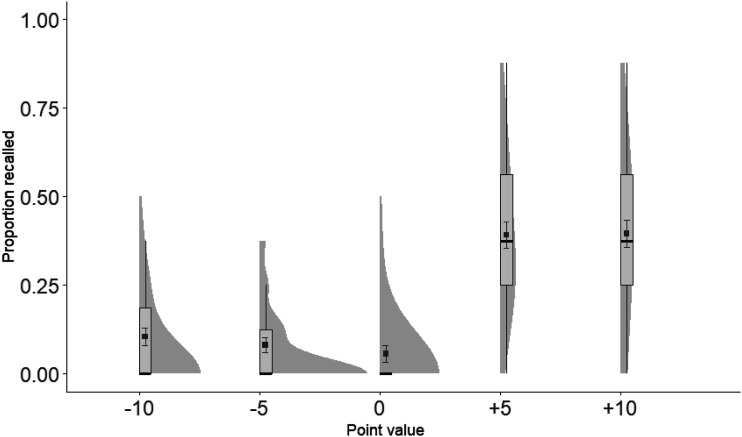
Proportion recalled plotted as a function of Point value in Experiment 2a. Elements include box-plots with medians and density clouds. Squares represent means, and error bars represent ± 1 standard errors. Analyses revealed superior recall for +5 and +10 point value associated words than words associated with other point values.

Proportion of recalled words were submitted to a one-way repeated measures ANOVA, with Point value (−10, −5, 0, +5, +10) as the independent variable. The ANOVA revealed a significant main effect, *F*(4, 120) = 31.97, *p* < .001, η_*p*_^2^ = .52 (see Figure 5). Planned *t* tests showed that both positive values differed from the neutral and the negative values, smallest *t*(30) = 5.97, all *p*’s < .001. There was no difference between the +5 and the +10 valued words, *t*(30) = .09, *p* = .932. There was also no difference between the −5 and −10 valued words, *t*(30) = .95, *p* = .351. Finally, there were no differences between the 0 words and either the −5 words, *t*(30) = 1.06, *p* = .296, or −10 words, *t*(30) = 1.93, *p* = .063.

#### Experiment 2b: Recognition

**Figure 6:**
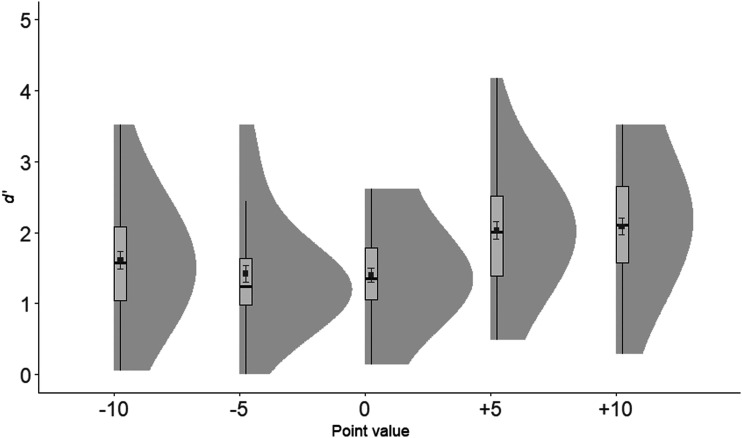
*d*′ plotted as a function of Point value in Experiment 2b. Elements include box-plots with medians and density clouds. Squares represent means and error bars represent ± 1 standard errors. Analyses revealed higher discrimination for +5 and +10 point value associated words than words associated with other point values, as well as higher discrimination for −10 than −5 and 0 point value associated words.

*d*′ values were submitted to a one-way repeated measures ANOVA, with Point value (−10, −5, 0, +5, +10) as the independent variable. The ANOVA revealed a significant main effect, *F*(4, 180) = 22.36, *p* < .001, η_*p*_^2^ = .33 (see Figure 6). Planned *t* tests showed that all the positive values differed from the neutral and the negative values, smallest *t*(45) = 3.63, all *p*’s < .001. There was no difference between the +5 and the +10 valued words, *t*(45) = .58, *p* = .568. *d*′ rates were significantly higher for −10 words than both −5 words, *t*(45) = 2.93, *p* = .021, and 0 words, *t* (45) = 3.01, *p* = .004, which was in contrast to the recall results in Experiment 2a. Again, there was no difference between the −5 and 0 valued words, *t*(45) = .70, *p* = .489.

### Discussion

In Experiment 2, participants learned a series of words, each associated with one of five possible point values (−10, −5, 0, +5, +10) that would ostensibly be received if the word was later retrieved. Before the test, participants were informed that the previously associated values were not meaningful, and they should attempt to retrieve all the words they learned. The results tended to support the conclusions from Experiments 1a and 1b, while also revealing several potentially intriguing findings that were unique to the graded value paradigm.

First, as in Experiments 1a and 1b, and in line with previous research (Knowlton & Castel, 2022), across both studies we found that memory for negatively valued words was inferior to memory for positively valued words. As mentioned above, the establishment of this effect is important as a baseline for comparing memory between neutrally and negatively valued words.

Second, in both Experiments 2a and 2b, we failed to find evidence of a graded VDR effect for positively valued words, as memory was equal for +5 and +10 words. While a graded effect of positively valued words is often (Ariel et al., 2015; Castel et al., 2009, 2011; DeLozier & Rhodes, 2015; Griffin et al., 2019; Murphy & Knowlton, 2022; Park et al., 2022; Robison & Unsworth, 2017; Stefanidi et al., 2018) but not always (Foster & Sahakyan, 2012, Experiment 3) found, the lack of the finding here may not be very surprising. A recent set of experiments (Filiz & Dobbins, 2024) showed that the graded effect is heavily dependent on encoding strategies. Given that learning in the current experiments was not iterated over multiple learning blocks and no instructions made explicit to the participants that they should focus mostly on the highest valued words (as has been shown to be a potentially important factor by Filiz & Dobbins, 2024), the participants may not have had enough opportunity to deploy differential encoding strategies based on the positive point values.

Third, Experiment 2b revealed an interesting pattern whereby memory for −10 words was higher than that for −5 and 0 words. One could potentially take this finding as evidence that a third memory strategy was being used, in which participants invested resources in remembering both positively and extreme negatively valued words. This strategy could be useful to remember negatively valued words, and their associated values, as to ensure that they are not reported on a later memory test. This interpretation should, however, be taken somewhat cautiously, as the results from Experiment 2a do not align with this notion, nor do the results of previous research which failed to find a graded retrieval pattern for negatively valued words (Castel et al., 2007; Friedman & Castel, 2011; Scholz & Dutke, 2019).

An alternative, but related and not mutually exclusive, explanation could be that salience of the ends of the *reward* scales are driving memory and that the −10 value is particularly salient in this case. This explanation would align with previous research that found a somewhat similar pattern of results using a related experimental paradigm with positive and negative values (Castel et al., 2016, Experiment 2), positive and neutral values (Castel et al., 2016, Experiment 1), and just positive values (Madan & Spetch, 2012). While future research should investigate this possibility in further detail, we would here, again, urge caution in overinterpreting our results as evidence of the role of salience in driving memory. For one, we do not find a clear graded pattern for positively valued words, as would be expected by this explanation, nor do we find the pattern for negative values in Experiment 2b mirrored in the results of Experiment 2a. A future investigation that attempts to address this explanation more explicitly could alter the current methods to bring them more in line with the iterative nature of past research (Castel et al., 2016; Madan & Spetch, 2012) and/or the learning or test types; these are all relevant factors that could influence the effect and manipulation of each could bear important insights into when graded, reversed graded, or U-shaped patterns may arise in the data.

Fourthly, and in line with the results from Experiments 1a and 1b, we found no evidence of poorer memory for negatively than neutrally valued words. In Experiment 2a, the recall rates of negative and neutrally valued words were not significantly different, though there were, again, concerns about floor performance on the recall test. Memory performance in Experiment 2b was clearly above chance, and again there was clearly no evidence of poorer memory performance in the negative conditions than in the neutral condition. The one difference was *better* performance in the −10 than −5 and 0 conditions, which is clearly at odds with a strategy that focuses on inhibiting (extreme) negative value words.

## Combined Analyses

While the results of all four experiments align more with a memory strategy that focuses on investing resources only in remembering positively valued words, and do not align with a memory strategy that also focuses on inhibiting memory for negatively valued words, one could argue that the power of the individual experiments was not high enough to find a smaller effect. Furthermore, while we failed to find evidence of poorer memory for negatively than neutrally valued words, this should not necessarily be interpreted as evidence in favor of the null hypothesis (i.e., that memory was equal between negatively and neutrally valued conditions; Anderson, 2020). To better interpret the results in terms of the null findings, we conducted additional Bayesian analyses, in which we also collapsed data across experiments with the same test type.

### Results

#### Data Preparation

For the combined analyses, all participants from the final samples Experiments 1a and 2a were combined to allow for one test of recall results and all participants from the final samples of Experiments 1b and 2b were combined to allow for one test of old/new recognition results. As our focus was mainly on determining whether there was any difference between negatively and neutrally valued words, the data were collapsed into three conditions: negative, neutral, and positive (see Table 2). All analyses were performed in JASP (JASP Team, 2022) with preset prior values being used. For both recall and recognition, we focused on the key test of interest, a directed (one-tailed) paired-samples Bayesian *t* tests between negative and neutral values.

**Table 2:** Description of which conditions were collapsed for the combined analyses

Combined analyses	Experiments 1a & 1b	Experiments 2a & 2b
Negative	−1	−10 & −5
Neutral	0	0
Positive	+1	+5 & +10
*Note*. Note that positive values were not used in the analyses reported here.		

#### Sensitivity Analyses

The sample size for the recall test in the combined analyses was *n* = 63, while the sample size for the recognition test was *n* = 80. Sensitivity analyses performed with G*Power (Faul et al., 2007) showed that, with the obtained sample sizes, and α and β levels of .05, we would have been able to detect a difference between the negative and neutral conditions of effect sizes *d*z = .42 (Recall) and *d*z = .37 (Recognition) using one-tailed *t* tests.

#### Recall

The paired samples *t* test revealed strong evidence in favor of the null hypothesis BF_0-_ = 13.90 that there was no difference in memory performance for negatively (*M* = 0.11, 95% credible interval: .08, .14) and neutrally (*M* = 0.09, 95% credible interval: .05, .13) valued words.

#### Recognition

The paired samples *t* test revealed strong evidence in favor of the null hypothesis BF_0-_ = 27.08 that there was no difference in memory performance for negatively (*M* = 1.49, 95% credible interval: 1.33, 1.64) and neutrally (*M* = 1.36, 95% credible interval: 1.21, 1.51) valued words.

### Discussion

When data were collapsed across experiments, both the recall and recognition data lead to the same conclusion. There is strong evidence that memory performance is not lower for negatively than neutrally valued words. These findings best align with the notion that participants use a memory strategy that focuses exclusively on investing resources in remembering positively valued words.

## General Discussion

Not all information that we encounter is equally relevant to encode and store in hopes of having it later available for retrieval. While the future relevance of information at the time it is encountered is not always known, and rarely completely known, there are frequently some cues as to what the future relevance of the information may be. In addition to knowing that retrieving some information may be associated with positive consequences, there are also cases where retrieving and acting on information in the future may be associated with negative consequences, or no consequences. In such situations, at least two memory strategies may be implemented, one in which resources are only invested in remembering information with positive consequences upon retrieval and behavior or a strategy which also focuses on inhibiting memory for information with negative consequences if retrieved and acted upon.

Across four experiments, we aimed to determine how words are remembered when associated with negative, neutral, and positive consequences if the words are reported on a memory test. We used a variety of the VDR paradigm (Castel et al., 2007) and tested memory with both free recall (Experiments 1a & 2a) and old/new recognition (Experiments 1b & 2b) tests. In line with previous research, we consistently found that memory for positively valued words exceeded memory for negatively valued words (Knowlton & Castel, 2022). Furthermore, we found that positively valued words were remembered better than neutrally valued words, which was in line with two previous studies (Foster & Sahakyan, 2012; Scholz & Dutke, 2019), but differed from another Lo (2021).

Both Foster and Sahakyan (2012) and Scholz and Dutke (2019) tested participants on their memory for single words that appeared sequentially in a learning phase (Foster & Sahakayan: auditorily; Scholz & Dutke: visually). This (visual) procedure was also used in the current experiments. By contrast, Lo (2021) tested associative memory and had participants try to associate a target picture with a background visual scene. During testing, participants were presented with the background scene and asked to choose which of three pictures was the one that was originally associated with the scene. Lo may have not found an enhancement in memory for positively valued items due either to the stimulus material (see Shaffer & Shiffrin, 1972 for a discussion of potential differences) used or the type of memory test used (e.g., Bisby & Burgess, 2014). Future research could be conducted to clear up these differences and test the generalizability of the superiority in memory for positively valued information.

Another consistent finding across all four of our experiments was that negatively valued words were not remembered worse than neutrally valued words. This was found regardless of whether we used free recall or recognition tests and regardless of whether we used a nongraded or a graded value paradigm. Additionally, when we combined data across multiple experiments, we found strong evidence in favor of the null hypothesis that memory performance is not lower for negatively than neutrally valued words. These findings align nicely with those of Scholz and Dutke (2019, Experiment 3) who found similar results with a recognition test using a graded value paradigm (though with different absolute values).

The lack of a difference between the negatively and neutrally valued words would be predicted by a memory strategy in which resources are invested only in remembering positively valued words. This strategy can possibly be compared to a selective rehearsal account of memory that is often considered to underly memory results found in item method directed forgetting paradigms (Bjork, 1970; Tan et al., 2020; see Tanberg et al., 2024 for a newer adaption of the theory).

In contrast to a strategy focused only on investing resources in remembering positively valued words, a strategy that also focused on inhibiting memory for negatively valued words would have predicted lower performance for negatively than neutrally valued words. This strategy could be compared to an inhibition (Fawcett & Taylor, 2008) or encoding suppression (Hubbard & Sahakyan, 2023) account of memory that has been used to explain findings from the item method directed forgetting paradigm. Evidence for this strategy being implemented has been found by Foster and Sahakyan (2012) who found worse memory for words associated with −5 points than words associated with 0 points.

Given the different findings in the literature, there is some confusion as to which memory strategy is most likely being used in situations where information is associated with later positive, neutral, and negative consequences upon retrieval and behavior. We would argue, however, that the evidence in favor of an account that focuses exclusively on investing resources in remembering positively valued information is stronger. For one, the current experiments and the experiment by Scholz and Dutke (2019), which support this account, are greater in number, greater in statistical power, and greater in methodological diversity than the evidence that supports an inhibition account (Foster & Sahakyan, 2012). Furthermore, the study by Foster and Sahakyan involves a comparison across different between-subjects conditions, without a direct comparison to positive values.

### Conclusions and Future Directions

To summarize, our findings across four experiments align well with the notion that a memory strategy is utilized in which resources are devoted only to the encoding of words associated with positive consequences upon retrieval and behavior. Our findings do not align with the notion that a memory strategy is utilized in which resources are devoted both to the encoding of words associated with positive consequences upon retrieval and inhibition of words associated with negative consequences upon retrieval.

Future research should focus more on possible graded effects of memory for items associated with various degrees of negative consequences upon retrieval. Doing so could allow for a fairer assessment of whether a memory strategy is implemented which focuses on devoting (encoding and retrieval) resources to remembering both positively and negatively valued information. Research could extend the range of possible negative consequences (such as in Castel et al., 2007), enhance the motivation by using real monetary incentives rather than arbitrary points (Adcock et al., 2006), use procedures that allow for a greater emphasis on the point values (Filiz & Dobbins, 2024; Schwartz et al., 2023), and use stimuli, number of stimuli, and test types that will help to avoid floor effects.

Another interesting avenue for future investigation in this area is the manipulation of stimulus material. In the current study, we used single item memory and (mostly) found no differences between memory for words with negative and neutral consequences upon retrieval. Lo (2021), who used paired-associates, similarly found no differences between negative and neutral items, but also found no differences to items associated with positive consequences upon retrieval. Future research could focus on the distinction between single item and associative memory, as well as on memory for different types of stimuli, such as pictures, sounds, or even odors. The use of different types of stimuli may allow for more specific interpretations of the strategies that are being used to invest resources in the memorization of items, as the strategies for remembering different types of material may not be the same (e.g., Shaffer & Shiffrin, 1972).

One further area that should be investigated in future research is memory for the source values associated with the words during the learning phase. While some research has shown evidence of value directed remembering effects without differential memory for the source values (e.g., Asfestani et al., 2020; Hennessee et al., 2017, 2019), more recent research has shown differential memory for source as a function of point value (Filiz & Dobbins, 2024). Future research which uses positive, neutral, and negative values and tests source memory for the values could be important in providing further evidence as to which memory strategy is likely being used by participants when faced with this paradigm.

A future focus on source memory could also help to clear up one final, and related, issue pertinent to the current research, namely the salience of the individual values used. It is unclear whether participants in the current experiments remembered the specific values associated with the target information or whether they remembered the gist of the values (e.g., whether an item was positive or not). As discussed above, this is an area of research in which the current empirical findings are varied (Filiz & Dobbins, 2024). By focusing on source memory for specific values, or including manipulation checks, future research can get a better grasp on how salient the different values are and how participants distinguish between the different values during encoding.

## Footnotes

^1^
This strategy can be compared to a selective rehearsal (e.g., Bjork, 1970) or a rapid retrieval check (Tanberg et al., 2024) theory of directed forgetting.

^2^
This strategy can be compared to retrieval inhibition (see MacLeod, 1998 for a review) or encoding suppression (Hubbard & Sahakyan, 2023) theories of directed forgetting where resources must be invested in order for items to be forgotten (Fawcett & Taylor, 2008).

^3^
Note that outlier removal criteria of 10% or below overall memory or not removing any participants both lead to the same pattern of results, including all reported significant results.

## Notes

**Conflict of Interest:** The authors declare that the research was conducted in the absence of any commercial or financial relationships that could be construed as a potential conflict of interest. There are no conflicts of interest in the conduct and reporting of research described in this article. Research reported in this article have not been published elsewhere, have not been posted on a listserv, and have not been shared on a website before submitting the manuscript.

**Publication Ethics:** No animal studies are presented in this manuscript. All procedures performed in our experiments involving human participants were in accordance with the ethical standards of the institutional and/or national research committees and with the 1964 Declaration of Helsinki and its later amendments or comparable ethical standards. Our participants were written informed about the experiment and their rights. They provided written informed consent. The experiments (including the treatment of participants) reported in this article were approved by the Local Ethical Committee of the Faculty 1 at the University of Hildesheim (Approval number 152). No potentially identifiable human images or data are presented in the manuscript.

**Authorship:** Ryan P.M. Hackländer, Helge Schlüter, Ann-Kathrin Rolke, Original conception & design; R.P.M. Hackländer, Simon Schuster, Programming; R.P.M. Hackländer, A. Rolke, S. Schuster, Data analyses; R.P.M. Hackländer, H. Schuster, S. Schuster,A. Rolke, Christina Bermeitinger, Data interpretation; R.P.M. Hackländer, H. Schlüter, S. Schuster, A. Rolke, C. Bermeitinger, Manuscript draft; R.P.M. Hackländer, H. Schlüter, S. Schuster, A. Rolke, C. Bermeitinger, Article revision.

**Open Science:** All information about the methods and results of the source memory tests, relevant summary data, as well as a discussion about the potential implications of findings and discussion of methodological concerns can be found on the associated OSF page: https://osf.io/u85vn/.

Open Data: The data that support the findings presented in this paper are openly available on Open Science Framework: https://osf.io/u85vn/. (Hackländer et al., 2025).

Pre-registration: Experiments 1b (https://osf.io/6mnxc) and 2b (https://osf.io/8p3tv) were preregistered on the Open Science Framework.
